# The effect of rollover footwear on pain, disability and lumbar posture in patients with low back pain

**DOI:** 10.1186/1757-1146-7-S1-A20

**Published:** 2014-04-08

**Authors:** Atefe Rahimi, Saeed Forghany, Christopher Nester, Fateme pol

**Affiliations:** 1 Musculoskeletal Research Centre, Isfahan University of Medical Sciences, Iran; 2 Centres for Health Sciences Research, University of Salford, UK

## Background

Low back pain (LBP) is one of the most common musculoskeletal disorders [1]. Exercise therapy is often advised [2,3] but requires a significant time commitment, can rely on equipment or health professionals and risks low compliance. As an alternative or adjunct, shoes with a curved sole profile are thought to produce beneficial changes in ankle, knee, hip and back position and posture [4]. Therefore, the aim of this preliminary study was to investigate the effect of rollover footwear on pain, disability and lumbar posture in patients with lumbar pain.

## Material and method

21 patients (age: 35.5±1.4 years) with LBP (pain distal to the buttocks that centralized with extension) were randomly assigned to a rollover footwear and lumbar extension exercise group (n=11) or a lumbar extension exercise only group (n=10). Baseline and 4 weeks post intervention measures were pain (visual analog scale), disability (Oswestry LBP disability) and lumbar posture when standing barefoot (as described by Forghany et al [4]).

Participants attended six appointments of 30 minutes duration over 4 weeks. Exercise consisted of 3 sets of 10 repetitions of extension exercises. Participants in the shoe and exercise group walked in rollover footwear [Perfect Steps] as often as possible over the 4 weeks (but at least 30 minutes each day).

## Results

Participants in both groups showed significant decreases in pain levels and disability after four weeks. Participants in the shoe and exercise group had significantly greater decreases in pain (p=0.04) and demonstrated 11.8% greater reduction in disability, but this did not reach statistical significance (p>0.05) (Table 1). The radius of lumbar curve was decreased in both groups when standing barefoot after 4 weeks, but not significantly (p>0.05) and there was no significant difference in the change in the radius of lumbar curve between two groups (p>0.05) (Table 1).

**Table 1:** Mean and amount of change in pain, disability and lumbar posture in exercise only, and exercise and shoe groups.

	Pain			Disability			Lumbar Posture (cm)		
	Pretest	4 weeks	change	Pretest	4 weeks	change	Pretest	4 weeks	change
Exercise	7.1±1.6	4.8±2.1^a^	-%31.5	36.9±10.5	26.3±9.5^a^	-%27.8	26.2±13.9	22.2±10.5	-%15.3
Exercise + shoe	7.3±1.8	3.0±2.0^a^	-%58.9^b^	29.8±5.3	18.0±5.9 ^a^	-%39.6	20.1±11.9	17.5±5.6	-%12.9

^a^ P <0.05 (within group). ^b^ P <0.05 (between group).

## Conclusion

This result suggests that the rollover footwear could be part of a treatment protocol for greater reduction in pain level in patients with LBP. However, the effects on lumbar biomechanics and association with changes in pain and disability remain unclear and requires further investigation.
